# Emergency management of profunda femoris artery rupture caused by subtrochanteric femoral fracture: A rare case report

**DOI:** 10.1097/MD.0000000000043497

**Published:** 2025-07-18

**Authors:** Bing Liu, Shengjie Wang, Zihao Liu, Guixian Dong

**Affiliations:** a Department of Traumatic Orthopedics, Hengshui City People’s Hospital, Harrison International Peace Hospital, Hengshui, Hebei Province, China.

**Keywords:** color Doppler, femoral fracture, profunda femoris artery

## Abstract

**Rationale:**

In clinical practice, subtrochanteric femur fractures are common. However, fractures accompanied by profunda femoris artery injury are relatively rare.

**Patient concerns:**

In our study, we report a case of a 50-year-old male admitted with a primary diagnosis of a right subtrochanteric femur fracture with rupture of the profunda femoris artery. The patient was admitted following a fall from a height.

**Diagnoses:**

Upon admission, X-ray showed a right subtrochanteric femur fracture, but physical examination revealed abnormal blood flow in the right groin area. A color Doppler ultrasound confirmed a rupture of the right profunda femoris artery.

**Interventions:**

During preparation for emergency surgery, the patient’s blood test results indicated severe anemia. We coordinated with the blood bank to provide a substantial blood supply to the patient, and successfully anastomosed the profunda femoris artery during surgery.

**Outcomes:**

Once vascular stability was achieved, we proceeded with intramedullary nailing fixation for the right subtrochanteric femur fracture, resulting in good fracture healing.

**Lessons:**

This newly identified injury pattern, subtrochanteric femur fracture with profunda femoris artery rupture, has not been previously reported in the literature. Further research is needed to explore the mechanism of this injury pattern, and it should be given due attention in clinical practice.

## 1. Introduction

Subtrochanteric fractures of the femur represent a specific category of intertrochanteric fractures, accounting for approximately 10% to 34% of femoral fractures.^[1]^ In younger patients, this injury is more prevalent in males and is often the result of high-energy trauma, such as falls from heights or vehicular accidents, typically accompanied by thoracic and abdominal injuries. In contrast, elderly patients, particularly females, frequently sustain this injury due to low-energy traumas, such as accidental falls.^[2]^ Vascular injuries associated with intertrochanteric fractures are exceedingly rare, with an incidence of 0.2%.^[3–5]^ Arterial damage may be iatrogenic, occurring during intramedullary fixation of intertrochanteric fractures caused by drill bits or distal locking screws breaching the medial cortex of the femur.^[3,4,6–9]^ Van Nguyen^[10]^ reported a case of femoral artery injury due to the steel wiring used in femoral stabilization. Isolated vascular injury due to fractures is exceptionally uncommon, and we have found no related reports. It remains unclear whether vessel rupture occurs immediately after the fracture or if chronic irritation from the fracture fragments leads to a pseudoaneurysm that subsequently ruptures.^[4,6–8,11–13]^ However, it is crucial to closely monitor vital signs in patients admitted for intertrochanteric fractures, looking for signs such as tachycardia, hypotension, thigh swelling, and rapid decline in hemoglobin levels.

A 50-year-old male patient with a subtrochanteric femoral fracture was admitted to the hospital. Upon admission, the laboratory results indicated severe anemia, and ultrasound examination revealed rupture of the profunda femoris artery. An emergency procedure was performed, including a vascular anastomosis of the ruptured profunda femoris artery. Once the vascular condition stabilized, surgical intervention was performed for the fracture. The patient showed good recovery.

## 2. Case report

**Figure 1.**
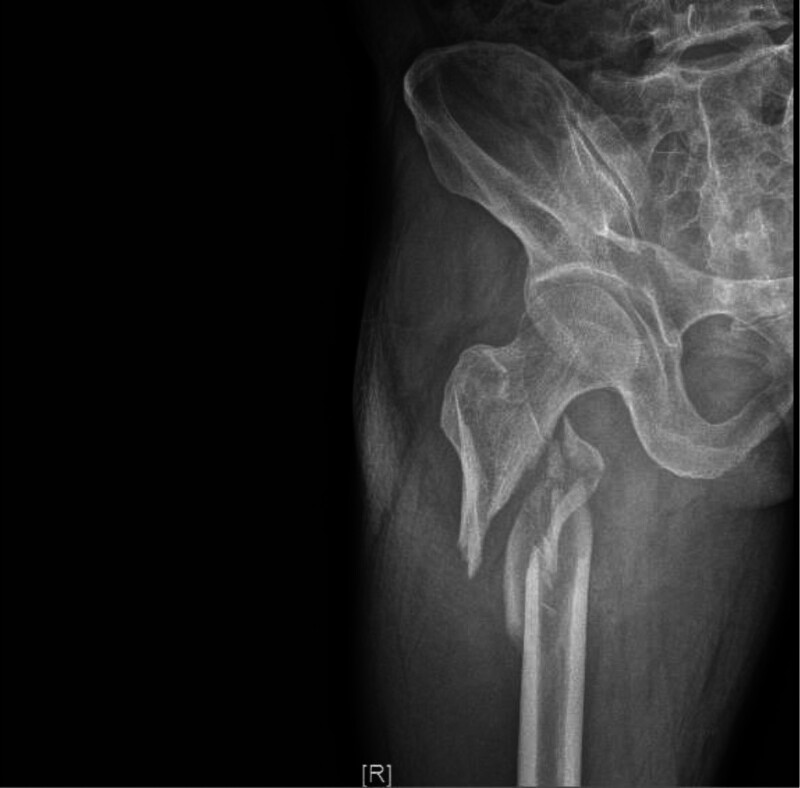
The X-ray after the injury shows a fracture below the femoral trochanter.

**Figure 2.**
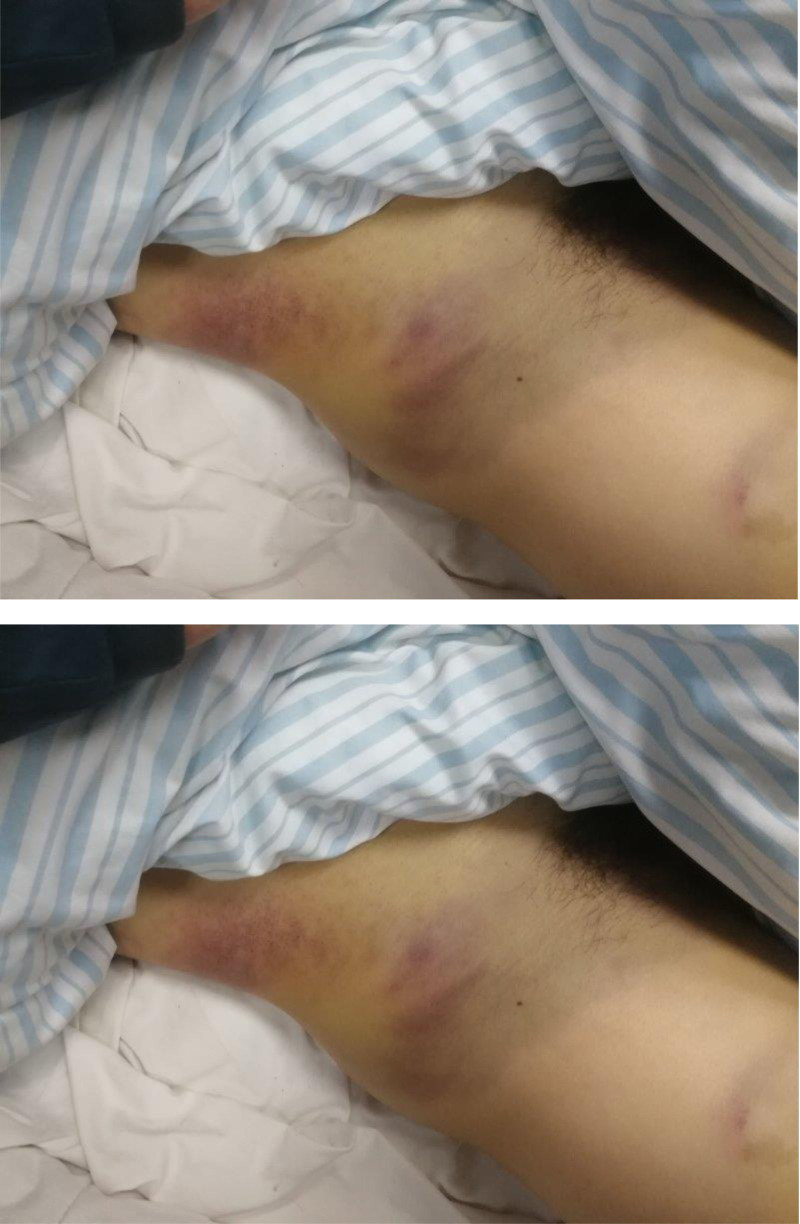
Swelling and bruising in the groin area.

**Figure 3.**
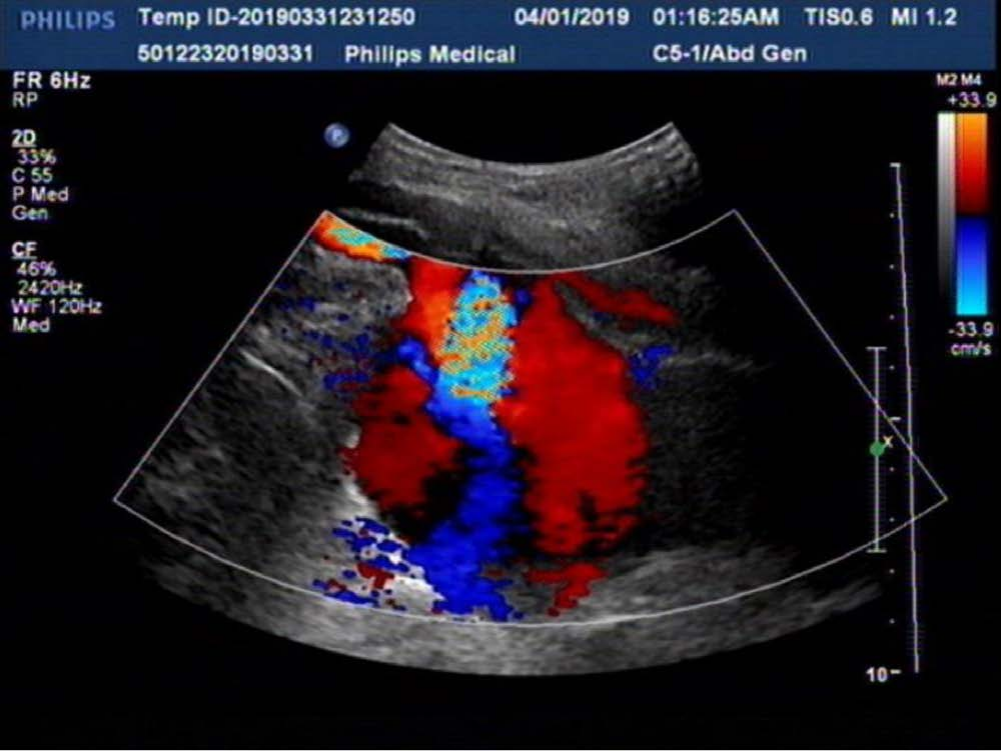
Color Doppler scans.

**Figure 4.**
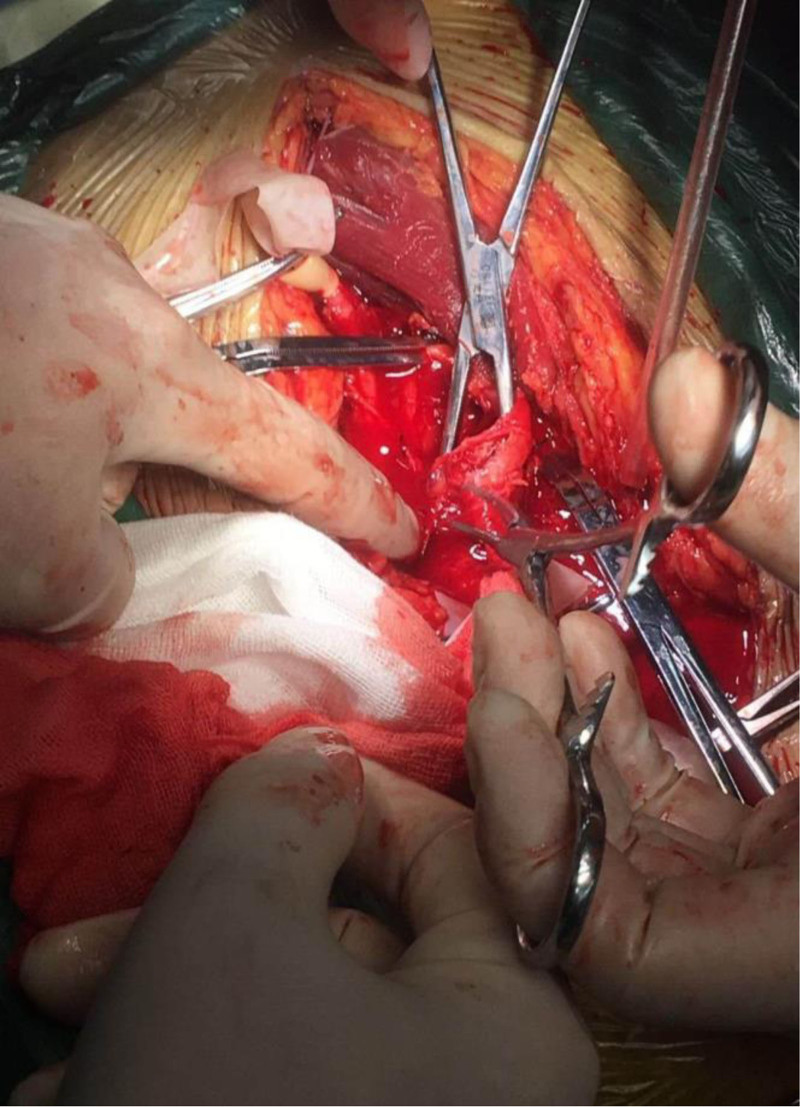
Rupture in the profunda femoris artery.

**Figure 5.**
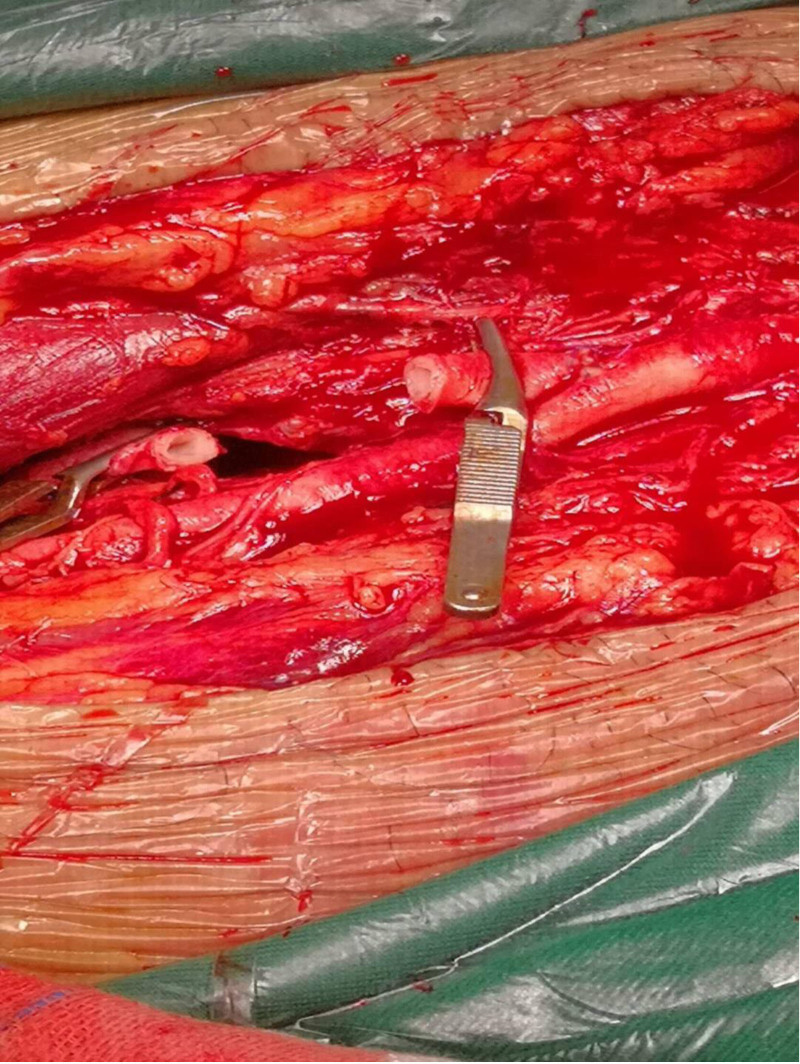
Clearing the ends of the blood vessels.

**Figure 6.**
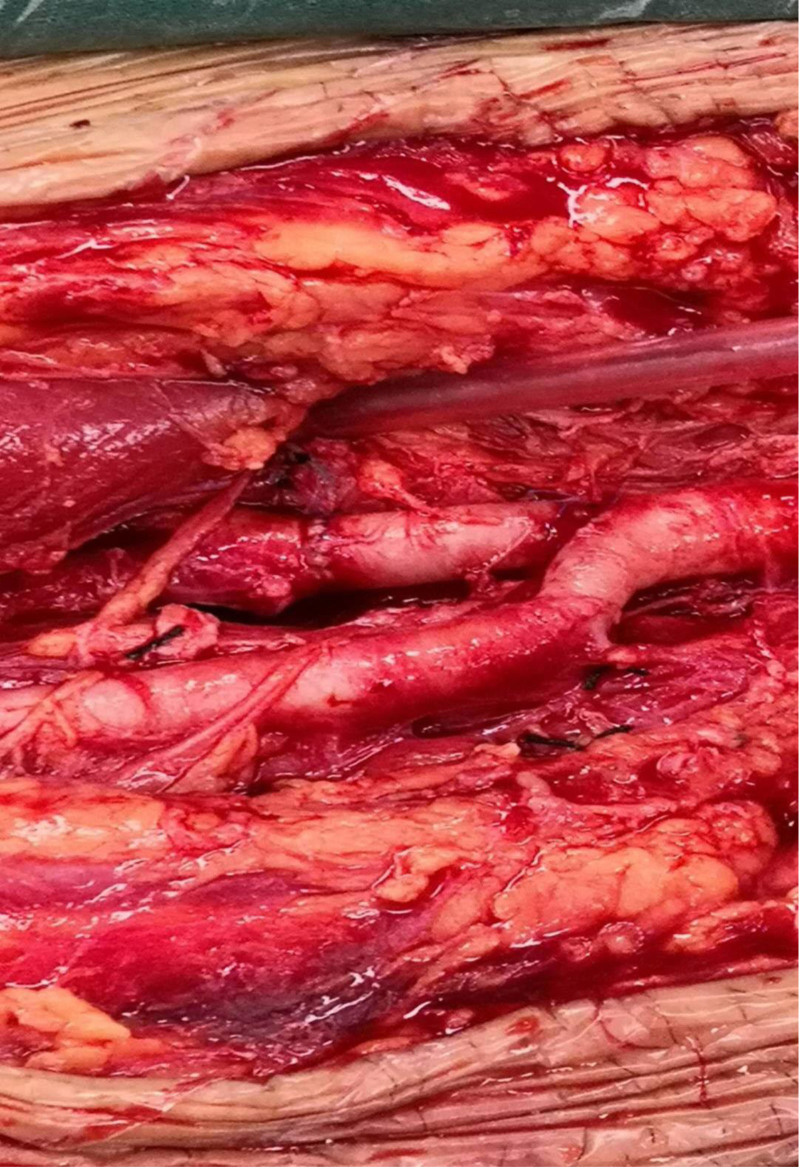
Image after vascular anastomosis.

**Figure 7.**
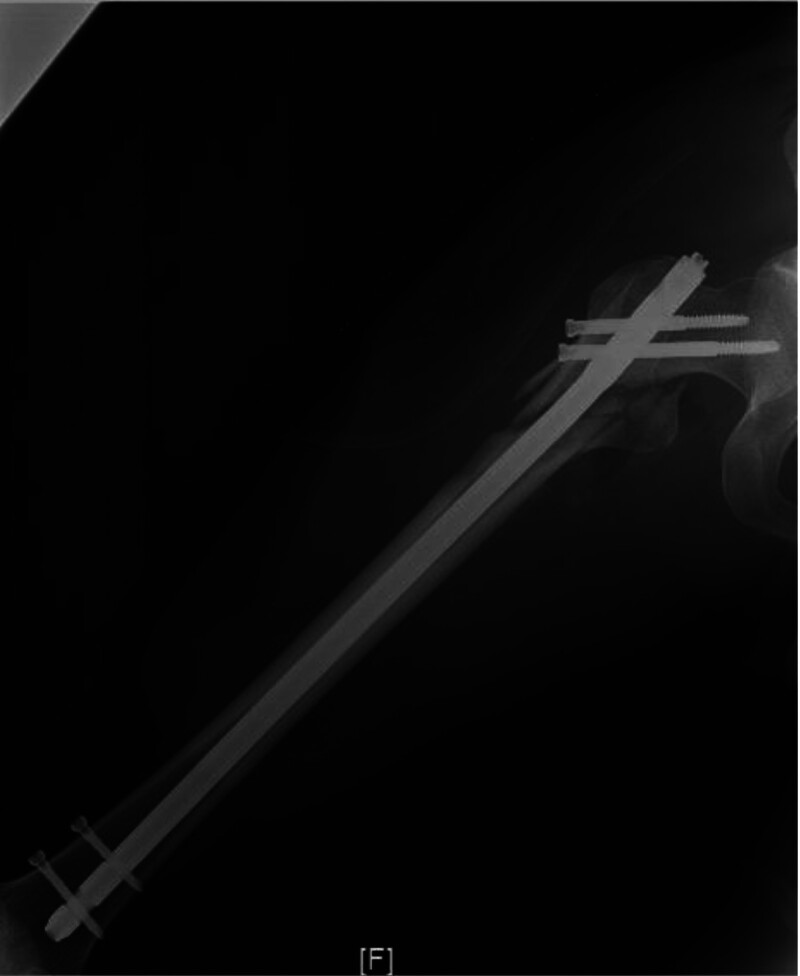
Postoperative internal fixation of a fracture.

**Figure 8.**
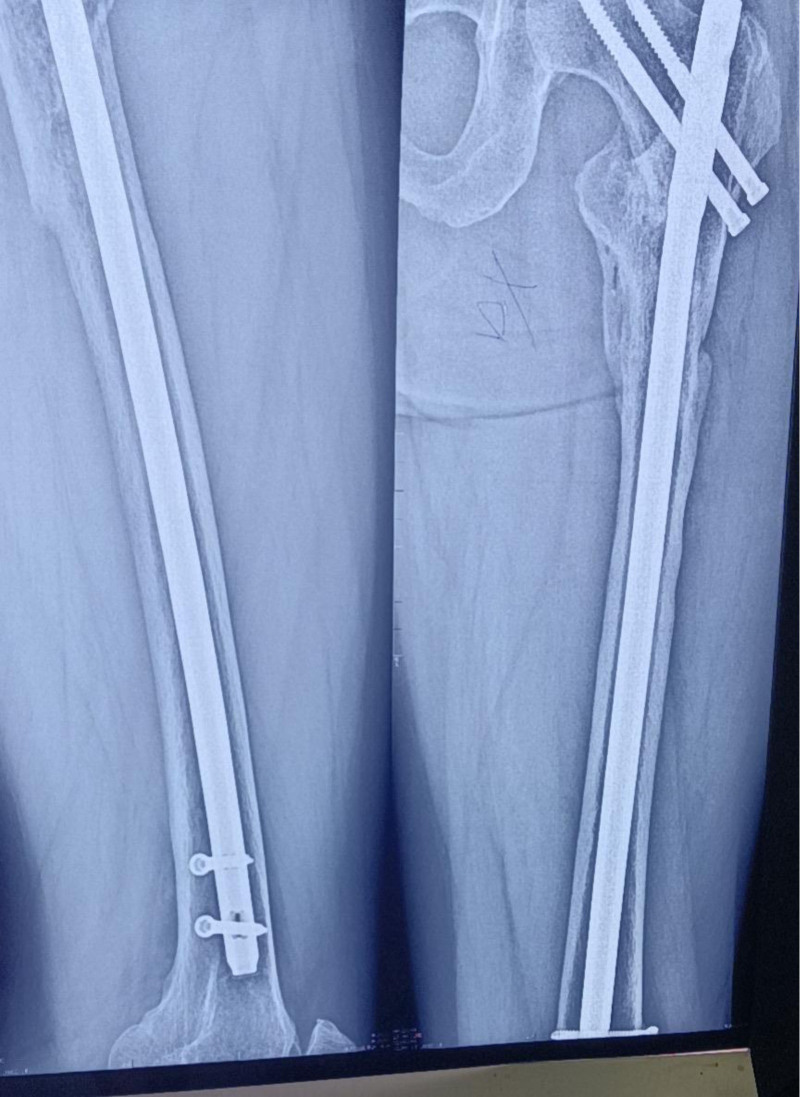
The patient underwent follow-up imaging at the local hospital.

A 50-year-old male patient fell from a height of 4 m while at work and was admitted to Wuyi County Hospital in Hengshui City, Hebei Province. The patient has an unremarkable past medical history, with no documented hypertension or diabetes mellitus, no chronic medication usage, no prior surgical procedures, and no known drug or food allergies. Imaging examination revealed multiple fractures of the right ribs and a fracture of the right femoral trochanteric region (Fig. 1). Three days later, he was transferred to our hospital, where the attending physician reported a daily decline in hemoglobin levels. Upon examination at our facility, the patient was found to have pale eyelids, extensive ecchymosis, and swelling in the groin area (Fig. 2). Palpation of the inguinal region revealed a pronounced wheezing sensation. Urgent blood tests showed a hemoglobin level of 36 g/L (normal range: 130–175 g/L) and platelet count of 43 × 10^9^/L (normal range: 100–350 × 10^9^/L). Clinical assessment and diagnostic studies in the Emergency Department strongly indicated femoral vessel disruption in the groin area. Prompt peripheral venous cannulation was initiated for volume replacement therapy concurrent with scheduling emergency vascular duplex ultrasonography. Color Doppler ultrasound of the right lower extremity blood vessels revealed a rupture of the right profunda femoris artery and the formation of a pseudoaneurysm (Fig. 3), immediately contacting the operating room to prepare for emergency surgery, and in parallel, reaching out to the blood bank for a substantial supply of blood. Following successful induction of anesthesia, the patient was positioned supine on the operating table with the right lower extremity slightly flexed at the hip and knee and externally rotated. A 25-cm longitudinal incision was made along the anatomical course of the right proximal femoral artery. Sequential dissection through the skin, subcutaneous tissue, and fascia revealed a substantial amount of serosanguinous fluid within the subcutaneous plane. The inguinal ligament was identified, and meticulous dissection of the vascular sheath exposed the femoral artery, which was subsequently clamped. Distal dissection revealed a 1-cm laceration at the axillary region of the femoral artery and the branch of the profunda femoris artery. The profunda femoris artery demonstrated a 1-cm full-thickness contused laceration between the medial and lateral circumflex femoral arteries, with vessel continuity maintained (Fig. 4). A subadventitial cavity was observed, and the femoral fracture end was palpable. The wound was thoroughly irrigated. Using microsurgical forceps, the adventitial connective tissue was dissected longitudinally along the vessel axis, mobilizing both ends of the vessel to facilitate a tension-free repair. The vessel ends were trimmed to ensure an intact intimal surface. The adventitia at the transected edge was retracted laterally with microsurgical forceps, and 0.5 to 1 mm of adventitia and perivascular tissue were excised to prevent intimal inversion. The vessel lumen was irrigated with heparinized saline to remove thrombotic material, confirming robust proximal inflow and unobstructed distal backflow. Microvascular clamps were applied 5 to 8 mm from each vessel end (Fig. 5). The vessel ends were approximated using a microvascular approximator, and end-to-end anastomosis was performed with 7-0 Prolene suture at 2 anchoring points (0° and 180°); 6 continuous sutures were placed on the anterior wall, and after rotating the vessel, 6 additional sutures were placed on the posterior wall. Upon release of the vascular clamps, distal arterial pulsation was restored, and no anastomotic leakage was observed (Fig. 6). The incision was closed in anatomical layers. A total of 10 units of red blood cells, 380 mL of plasma, 10 doses of cryoprecipitate, and 1 therapeutic dose of platelets were administered intraoperatively. Postoperatively, the patient’s vital signs were stable, and routine blood tests conducted on postoperative days 1, 2, 3, and 6 showed hemoglobin levels of 90, 90, 93, and 95 g/L, respectively. The platelet counts were 52 × 10^9^/L, 66 × 10^9^/L, 77 × 10^9^/L, and 102 × 10^9^/L. Surgical treatment for closed reduction and internal fixation of the fracture below the greater trochanter of the right femur was performed days later (Fig. 7). Serial hematological analyses were performed following surgical intervention. Postoperative hemoglobin monitoring demonstrated values of 88, 87, 89, and 92 g/L on postoperative days 1, 2, 3, and 6, respectively. Thrombocyte counts showed progressive improvement from 99 × 10^9^/L to 123 × 10^9^/L over the same period. CRP levels exhibited a downward trend from 43.32 to 5.15 mg/L, indicating resolution of the acute phase response. The patient remained afebrile throughout the postoperative course. Implemented concurrent therapeutic mobilization protocol. The initial phase commenced on the first postoperative day with ankle dorsiflexion/plantarflexion exercises and quadriceps isometric contractions. By the third postoperative day, patient was positioned at 45° elevation with guided active-assisted range of motion exercises focusing on knee flexion-extension. At 1-week postprocedure, initiated partial weight-bearing ambulation with assistive devices, maintaining <10 kg load on affected right lower extremity. Exercise progression was titrated according to patient’s physiological response, adhering to established rehabilitation protocols. Functional assessment via Harris Hip Score demonstrated progressive improvement: 43 points at 1-month follow-up, advancing to 71 points at 2-month evaluation. Patient achieved independent ambulatory status without assistive devices by month 3. Complete functional recovery with return to activities of daily living and occupational duties was documented at 7-month follow-up. As the patient’s residence is quite far from me, the patient will send the results to my hospital after undergoing a follow-up examination at the local hospital (Fig. 8). The patient’s fractures healed well.

Throughout the admission period, I maintained enhanced therapeutic communication with the patient, conducting active listening assessments and monitoring their psychosocial status. I provided psychological support and reassurance to mitigate anxiety and improve treatment adherence. Concurrent psychological intervention protocols were implemented, with licensed clinical psychologists conducting structured therapeutic sessions to address maladaptive psychological responses. Treatment efficacy was reinforced through evidence-based outcome demonstrations. The patient’s support network was optimized through comprehensive caregiver education regarding pharmacological management, nutritional requirements, assisted ambulation protocols, and rehabilitative interventions.

Given the presence of vascular compromise, we initiated subcutaneous administration of low molecular weight heparin calcium at a dosage of 4100 AXaIU to mitigate the risk of thromboembolic events. To prevent complications such as fracture displacement, hardware failure, and avascular necrosis, early full weight-bearing and excessive active mobilization of the affected extremity were strictly contraindicated. Concurrently, to reduce the risk of delayed union, a graduated weight-bearing regimen was employed to promote callus formation at the fracture site. Follow-up assessments demonstrated marked improvement in the patient’s posttraumatic anxiety. At 7 months postinjury, the patient successfully returned to work without any sequelae related to the initial trauma.

## 3. Discussion

Femoral vessel disruption concurrent with proximal femoral and hip fractures demonstrates remarkably low prevalence, with documented occurrence in 0.2% of cases.^[3–5]^ Contemporary literature suggests this reported incidence of vascular compromise predominantly reflects iatrogenic etiology secondary to operative fixation interventions.^[3,4,6,8,13]^ Vascular injury to the profunda femoris artery secondary to femoral fractures is an exceptionally uncommon complication. Ge et al documented a case involving a femoral shaft fracture complicated by profunda femoris artery laceration. Preoperatively, the patient exhibited sustained hypotension and hypoxemia. Computed tomography angiography identified a rupture of the profunda femoris artery.^[14]^ According to Murphy et al,^[6]^ in cases of arterial injury resulting from hip fractures, most patients experience spontaneous thrombosis, which leads to resolution. However, in this patient, there was ongoing vascular bleeding days after injury to the profunda femoris artery, resulting in severe anemia.

The diagnosis of arterial injury following proximal femoral fractures, both postinjury and postsurgery, relies primarily on the clinical presentation of the patient. It is crucial for clinicians to maintain vigilance and to combine this information with appropriate imaging examinations. The evaluation of arterial injury should include complete blood count, color Doppler ultrasound of the swelling site, and angiography. Owing to its advantages of convenience, safety, and noninvasiveness, color Doppler ultrasound is preferred as the initial examination for diagnosing hematomas or pseudoaneurysms. Computed tomography angiography is a rapid, precise, and intuitive technique with a sensitivity of 96.2% and specificity of 99.2% for diagnosing arterial injuries. It can visualize the anatomical structure of blood vessels from multiple angles while also revealing the condition of the surrounding structures, thereby providing valuable reference points for clinicians to develop treatment strategies.^[15]^ However, in the case of our patient, given the urgency of the situation, we could only opt for the less time-consuming color Doppler ultrasound.

## 4. Conclusion

In this case, we observed rupture of the profunda femoris artery following a subtrochanteric femur fracture, resulting in significant blood loss and anemia. Previous studies have reported arterial injury as a complication of fracture surgery. Therefore, we recommend that for patients with subtrochanteric femoral fractures, clinicians should enhance physical examinations and conduct thorough laboratory monitoring to prevent severe complications due to vascular injuries, which could even threaten the patient’s life.

## Author contributions

**Conceptualization:** Bing Liu.

**Data curation:** Bing Liu.

**Writing – original draft:** Bing Liu.

**Investigation:** Shengjie Wang.

**Formal analysis:** Zihao Liu.

**Writing – review & editing:** Guixian Dong.
